# Prognostic and predictive role of CD8 and PD-L1 determination in lung tumor tissue of patients under anti-PD-1 therapy

**DOI:** 10.1038/s41416-018-0220-9

**Published:** 2018-10-15

**Authors:** Jean-David Fumet, Corentin Richard, Fanny Ledys, Quentin Klopfenstein, Philippe Joubert, Bertrand Routy, Caroline Truntzer, Andréanne Gagné, Marc-André Hamel, Camila Figueiredo Guimaraes, Bruno Coudert, Laurent Arnould, Laure Favier, Aurélie Lagrange, Sylvain Ladoire, Pierre Saintigny, Sandra Ortiz-Cuaran, Maurice Perol, Pascal Foucher, Paul Hofman, Marius Ilie, Sandy Chevrier, Romain Boidot, Valentin Derangere, François Ghiringhelli

**Affiliations:** 1 Department of Medical Oncology, Center GF Leclerc, Dijon, France; 2 Research Platform in Biological Oncology, Dijon, France; 3 GIMI Genetic and Immunology Medical Institute, Dijon, France; 4 University of Burgundy-Franche Comté, Dijon, France; 5 Institut Universitaire de Cardiologie et de Pneumologie de Québec, Laval University, Quebec City, QC Canada; 6 Centre de Recherche du Centre Hospitalier de l’Université de Montréal (CRCHUM), Montréal, QC Canada; 7 Hematology–Oncology Division, Department of Medicine, Centre Hospitalier de l’Université de Montréal (CHUM), Montréal, QC Canada; 8 Department of Pathology, Center GF Leclerc, Dijon, France; 9 Department of Medical Oncology, Center Leon Berard, Lyon, France; 10 Department of Thoracic Oncology, Dijon University Hospital, Dijon, France; 11 Laboratory of Clinical and Experimental Pathology, FHU OncoAge, Nice University Hospital, Université Côte d’Azur, Nice, France; 12 Hospital-Integrated Biobank (BB-0033-00025), FHU OncoAge, Nice University Hospital, Université Côte d’Azur, Nice, France; 13 INSERM UMR1231, Dijon, France

**Keywords:** Predictive markers, Tumour biomarkers

## Abstract

**Background:**

No study has evaluated the predictive and prognostic role of CD8 and PD-L1 coexpression in non–small-cell lung cancer (NSCLC).

**Methods:**

We analyzed RNA sequencing and/or immunohistochemistry staining in NSCLC patients from The Cancer Genome Atlas (*n* = 1016), and 34 metastatic NSCLC samples not treated by immunotherapy as prognostic cohorts. As predictive aspect of CD8 and PD-L1, we used 85 NSCLC patients treated with anti-PD-1. Two validation cohorts were used including 44 NSCLC patients treated with anti-PD-1 and an external cohort with different tumor types.

**Results:**

In prognostic cohorts, high *CD8A* expression was associated with longer OS (*p* = 0.02), while high *CD274* mRNA was associated with poor prognosis (*p* = 0.05). In predictive cohort, high CD8 expression and *CD8A* mRNA were associated with longer progression-free survival (PFS) (*p* = 0.0002). There was no significant association between PD-L1 expression and PFS while high *CD274* mRNA was associated with longer PFS (*p* = 0.009). A combination of CD8A and CD274 was highly predictive of outcome. These results were confirmed in the validation cohorts. This two-genes signature demonstrated similar results compared to gold standard signatures.

**Conclusion:**

CD8 represents both a prognostic and predictive factor of outcomes, while PD-L1 share different prognostic and predictive roles.

## Background

Lung cancer is currently the most common type of cancer, and it accounts for the highest number of cancer deaths worldwide. Approximately, 80 % of newly diagnosed cases of non–small-cell lung cancer (NSCLC) are inoperable with locally advanced or metastatic disease.^1^ The immune system controls NSCLC progression, and CD8^+^ infiltrates are associated with better outcome.^2^ Programmed cell death-ligand-1 (PD-L1), the ligand of PD-1, is expressed in many cancer tissues, including NSCLC.^3,4^ The identification of PD-L1 as a distal immune modulator of adaptive CD8^+^ T-cell anticancer response was at the origin of the development of monoclonal antibodies targeting PD-1 (nivolumab and pembrolizumab) or PD-L1 (atezolizumab and durvalumab). These immune checkpoint PD-1/PD-L1 blocking agents showed remarkable clinical efficacy with long lasting clinical response in typically immunogenic tumors such as melanoma, renal cell carcinoma, bladder cancer, and both squamous cell carcinoma and nonsquamous NSCLC.^5–13^

Therefore, PD-1/PD-L1 pathway targeted immunotherapy has become a standard option for the management of locally advanced and metastatic lung cancer.^5,6,8,9^ In clinical trials, anti–PD-1 and anti–PD-L1 antibodies produced a durable response in approximately 20% of unselected patients with advanced NSCLC in the second or third lines of treatment.^8,9^ There is no optimal predictive biomarker to select patients that would likely respond to anti-PD-1/PD-L1 therapies. Developing validated biomarkers remains a major challenge.

While genomic marker like tumor mutation burden becomes promising (Cancer Cell. 2018 May 14;33(5):843–852), PD-L1-expression represents the only clinically available and approved marker to predict response to PD1/PD-L1 axis inhibitors.^5–7,14^ In first line therapy of advanced NSCLC, pembrolizumab, demonstrated its superiority in comparison to chemotherapy in patients with high PD-L1 expression (>50%).^5^ Surprisingly, the similar test could not predict response to nivolumab, in a similar setting.^14^ Indeed, although the expression of PD-L1 may potentially serve as a predictive biomarker to identify patients that respond to treatment, it remains an imperfect biomarker. Subsequent work has shown that patients with PD-L1-negative tumors showed an aggregate 15% response rate (RR) across many types of cancer.^15^ PD-L1 expression used as a predictive marker has a poor negative predictive value, therefore selection of patients using only PD-L1 may exclude potentially responding patients. In contrast to the large amount of data on the predictive role of PD-L1 expression, its prognostic role remains unclear.

Additional strategies are being developed to predict clinical response to PD-1 checkpoint blockade. Transcriptomic signatures related to inteferon (IFN)-γ signaling and activated T-cells are currently validated across tumors in patients treated with pembrolizumab.^16,17^ In addition, in the case of insufficient tumor-infiltrating lymphocytes (TILs) in a tumor, it is unlikely that PD-1/PD-L1 blockade would lead to a specific T-cell response^18^ despite PD-L1 expression. CD8^+^ T cells, which are the principal cytotoxic cells, are pivotal for cell-mediated antitumor immune responses. A study by Tumeh et al.^19^ found that preexisting CD8^+^ T cells are essential for tumor regression following therapeutic PD-1/PD-L1 blockade in metastatic melanoma, indicating that CD8^+^ TILs play a key role in anti-PD-1 therapy response. Recently, Teng et al.^20^ proposed to classify tumors into four groups based on PD-L1 and CD8 expression. They defined four categories: type I adaptive immune resistance (PD-L1 positive and high TILs), type II immune ignorance (PD-L1 negative and low TILs), type III intrinsic induction (PD-L1 positive and low TILs), and type IV immune tolerance (PD-L1 negative and high TILs) pathways. However, there is no description of the predictive and prognostic roles of CD8 and PD-L1 coexpression in NSCLC patients receiving immune checkpoint inhibitors. The first aim of this study was to evaluate the prognostic role of PD-L1 and CD8 mRNA and protein expression in patients with NSCLC. The second aim was to evaluate the predictive role of PD-L1 and CD8 mRNA and protein expression in patients with NSCLC treated with nivolumab after failure of platinum-based chemotherapy.

## Materials and methods

### Patient characteristics

#### Prognosis immunohistochemistry cohort

We, retrospectively, reviewed 34 patients with stage IIIB-IV NSCLC who received at least one line of platinum-based therapy, but no immunotherapy. The median age of patients at the first-line therapy was 65 years. Two-thirds of patients had nonsquamous cell cancer. All clinical characteristics are shown in Supplementary Table 1.

#### Prognosis mRNA cohort

For The Cancer Genome Atlas (TCGA) RNA-seq prognostic cohort (Supplementary Table 2), raw data from 1016 samples was downloaded using the TCGA2STAT software package for statistical analysis in R,^21^ which includes the lung adenocarcinoma cohort and the lung squamous cell carcinoma cohorts.

#### Predictive nivolumab cohort

We, retrospectively, reviewed 85 patients from three French university hospitals with stage IIIB or IV NSCLC who had previously received one or two lines of chemotherapy. All patients were treated with first-line platinum-based chemotherapy. None of the patients had epidermal growth factor receptor, anaplastic lymphoma kinase, B-Raf proto-oncogene serine/threonine kinase, or ROS1 oncogenic driven tumors. Upon progression, they received 3 mg/kg nivolumab administered intravenously as a single agent every two weeks. Tumor response was evaluated by computed tomography (CT) scan every four cycles. Response evaluation criteria in solid tumors (RECIST) 1.1 was used to define objective clinical response. The median age of patients at the introduction of nivolumab was 63 years. The most predominant histological type was adenocarcinoma (53%), followed by squamous cell carcinoma (44%). Clinical characteristics are shown in Supplementary Table 3. Tumors were collected, stored, and used with informed, written consent from the patients. The study was performed in accordance with the guidelines of the Declaration of Helsinki.

We successfully performed CD8 and PD-L1 assessments using immunohistochemistry (IHC) for 78 patients, and RNA sequencing for 43 patients. Other patients were not analyzed because of lack of sufficient biological material or low quality RNA.

#### Validation predictive cohort

We, retrospectively, reviewed 44 patients from Institut Universitaire de cardiologie et de pneumologie de Québec, with stage IIIB or IV NSCLC. All patients were treated in first line by pembrolizumab or further lines by nivolumab. Clinical characteristics are shown in Supplementary Table 4.

### IHC procedures

Thin tissue sections measuring 4 µm were cut from formalin fixed paraffin embedded specimen. All IHC procedures were performed using Benchmark apparatus (Ventana) and diaminobenzidine (DAB) as chromogenic revelator. All antibodies and secondary reagents used are reported in Supplementary Table 4. Once stained and permanently mounted, slides were digitalized with Nanozoomer HT2.0 (Hamamatsu) at 20× magnification and two pathologists independently analyzed numerized files. Scores were compared and when discrepancies occurred a third pathologist reviewed the slides and solved the results.

PD-L1 expression was evaluated with Sp142 clone (SpringBio, Ventana) based on scores determined by Poplar study.^11^ Briefly, scores were achieved as a percentage of tumor cells expressing PD-L1: TC3 > 50%, TC2 > 5% and <50%, TC1 > 1% and <5%, TC0 < 1%; tumor infiltrating immune cells were also scored as a percentage of tumor area: IC3 > 10%, IC2 > 5% and <10%, IC1 > 1% and <5%, IC0 < 1%. So, an IC and/or TC score of two or more was considered as a high expression.

22C3 clone (Dako) was also used to evaluate PD-L1 expression. Results obtained with this clone confirmed our previous evaluations achieved with Sp142 clone. For this antibody, stain was restrained to tumor cells and scores were evaluated as a percentage of tumor cells expressing PD-L1 as Scheel et al.^22^ with a three-bins method: TC < 1%, TC > 1% and <50%, TC > 50% (Figure S1A).

Cytotoxic T cells infiltrates were evaluated using CD8 labeling with C8/144B clone (Dako). Number of positive cells per area (µm^2^) was obtained using the recently released QuPath free software designed by Peter Bankhead.^23^ Briefly, two pathologists manually selected a CD8^+^ hot spots area of approximately 1 mm^2^ on digitalized slides in a blind fashion. Then, within this area, nuclei were detected and CD8^+^ cells were determined with a DAB threshold using automated Qupath algorithms (Figure S1B). The mean of the two values was used. In case of more than 5% of discordance, the two pathologists performed a second lecture jointly. The same algorithms (nuclei and DAB detections) were used for every slide to have homogenized results.

### RNA sequencing analysis

Total RNA was extracted from formalin-fixed paraffin-embedded (FFPE) tumor slices (5 × 5 µm) using the Maxwell 16 LEV RNA FFPE Purification kit (Promega) following manufacturer instructions. Libraries were prepared from 12 µl of total RNA with the TruSeq Stranded Total RNA using Ribo-Zero (Illumina) following manufacturer instructions. Once qualified, paired-end libraries were sequenced using 2 × 75 bp output on a NextSeq 500 device (Illumina).

The abundance of transcripts from RNA-seq data was quantified through the Kallisto program.^24^ This program is based on pseudo alignment for rapidly determining the compatibility of reads with targets, without the need for alignment. The Kallisto transcript index used as reference was built from merged human cDNA and ncDNA files from GRCh37 assembly ENSEMBL. Gene-level count matrices were then created with the DESeq2 library. Low-count genes were pre-filtered by removing genes with too few reads.^25^ Enrichr software was used to analyze Kyoto Encyclopedia of Genes and Genomes (KEGG) pathways.^26,27^

### Statistical methods

Data analysis was performed using R statistical software (http://www.R-project.org/) and presented with Prism 7 (GraphPad, San Diego, CA, USA). All patients from the prognostic cohorts were followed up until death or the end of data recording (November 1, 2017).

For the prognostic IHC cohort, composed with stage IIIB-IV patients, overall survival (OS) was calculated from the day the metastatic cancer was diagnosed to the date of death (all causes). Survivors were censored after 12 months. For the TCGA prognostic mRNA cohort, composed with patients from all stages (I–IV), OS was used for the survival analysis. Survivors were censored after 120 months. RNAseq gene expressions were based on RSEM raw counts normalized using variance stabilizing transformation in DESeq2 R package.^25^ For the predictive cohort, treatment RR was determined from CT scan analysis according to RECIST 1.1 (progression disease (PD); stable disease); partial response (PR))^28^ after 2–3 months of therapy. Progression-free survival (PFS) was defined as the time from the first day of treatment to the first recorded evidence of disease progression according to RECIST, clinical evaluation or death. For PFS, survivors were censored after 6 months.

Associations between disease characteristics and RR (PR + SD versus PD) were tested using chi^2^ or Fisher’s exact tests for qualitative variables and the Mann–Whitney test for continuous variables, as appropriate. All boxplots were drawn with a median, quartiles and Tukey’s whiskers.

Univariate Cox proportional-hazards models of all clinical and biological baseline variables were built to estimate hazard ratios (HRs) with a 95% CI. The best cutoff points for continuous variables were chosen using Cutoff Finder.^29^ Survival curves were estimated using the Kaplan–Meier method and compared using log-rank tests. Median follow-up was calculated using the reverse Kaplan–Meier method or recorded as not reached (NR) as appropriate. Multivariate models for PFS were created including sex, age (continuous), World Health Organisation (WHO) performance status and histology if available, and biological parameters (CD8, PD-L1). The predictive power of these models was compared using Harrell’s C index and the goodness of fit was estimated using Akaike Information Criterion (AIC). The two final multivariate models combining expression of CD8 and PD-L1 measured by mRNA or IHC were internally validated using bootstrapping (200 replications).

Differential gene expression analyses were performed using the DESeq2 R package.^25^ A gene was considered as differentially expressed if the false discovery rate-adjusted *p* value of the corresponding test was less than 0.05.

## Results

### Prognostic role of CD8 and PD-L1 expression in the IHC prognosis cohort

**Fig. 1:**
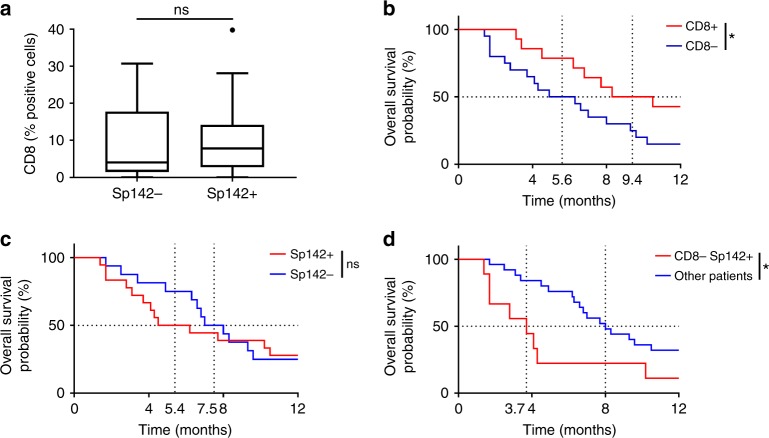
Prognostic role of CD8 and PD-L1 expressions in a control cohort of metastatic NSCLC using IHC. **a** Box plots showing the CD8 IHC expression respectively for patients with high (Sp142+) or low (Sp142−) expression of Sp142 labeling. **b**, **c** Kaplan–Meier estimates for overall survival; patients were stratified according to the CD8 (**b**), and Sp142 (**c**) labeling expressions: high expression (CD8+/Sp142+; in red) and low expression (CD8−/Sp142−; in blue). **d** Kaplan–Meier estimates for overall survival; patients were stratified in two groups: a group with low expression of CD8 labeling (CD8−) and high expression of Sp142 labeling (Sp142+) and a group with the other patients. Cutoffs for low and high expressions were defined with the Cutoff Finder method. ^*^p < 0.05; ^**^p < 0.01; ^***^p < 0.001; ****: *p* < 0.0001; ns not significant

In 34 cases of metastatic NSCLC treated without immunotherapy, we tested the prognostic role of CD8^+^ TILs and PD-L1 IHC expression. We observed a high concordance between 22C3 and Sp142 labeling (Fisher’s Exact test *p* = 0.0001) (Supplementary figures 2A, B). Sp142 mAb was then used for PD-L1 assessment. CD8^+^ TILs and PD-L1 IHC expression were not associated (Wilcoxon signed-rank test sum *p* = 0.46) (Fig. 1a). We observed that high CD8^+^ TILs IHC expression was associated with better OS (9.4 months versus 5.6 months; log-rank test *p* = 0.05) (Fig. 1b). OS did not differ between patients with high PD-L1 IHC expression and those with low PD-L1 IHC expression values (5.4 months versus 7.5 months; log-rank test *p* = 0.79) (Fig. 1c). Furthermore, according to Teng’s classification high PD-L1/low CD8^+^ TILs tumors are suspected to have most deleterious immune microenvironment and potentially the worst prognosis. Indeed, we observed that patients with low CD8 expression and high PD-L1 expression had very poor outcomes, with a median OS of 3.7 months, while the median for all other patients was 8 months (log-rank test *p* = 0.02) (Fig. 1d). In a multivariate Cox proportional model including age, sex, performance status, and histology, we observed a trend for poor outcomes in patients with low CD8^+^ TILs and high PD-L1 IHC expression (Supplementary Table 5).

Together these data underline that among patients with metastatic NSCLC not treated with immune checkpoint inhibitors, those with high PD-L1 and low CD8 TILs IHC have a poorer prognosis.

### Prognostic role of CD8 and PD-L1 expressions in the mRNA prognosis cohort

**Fig. 2:**
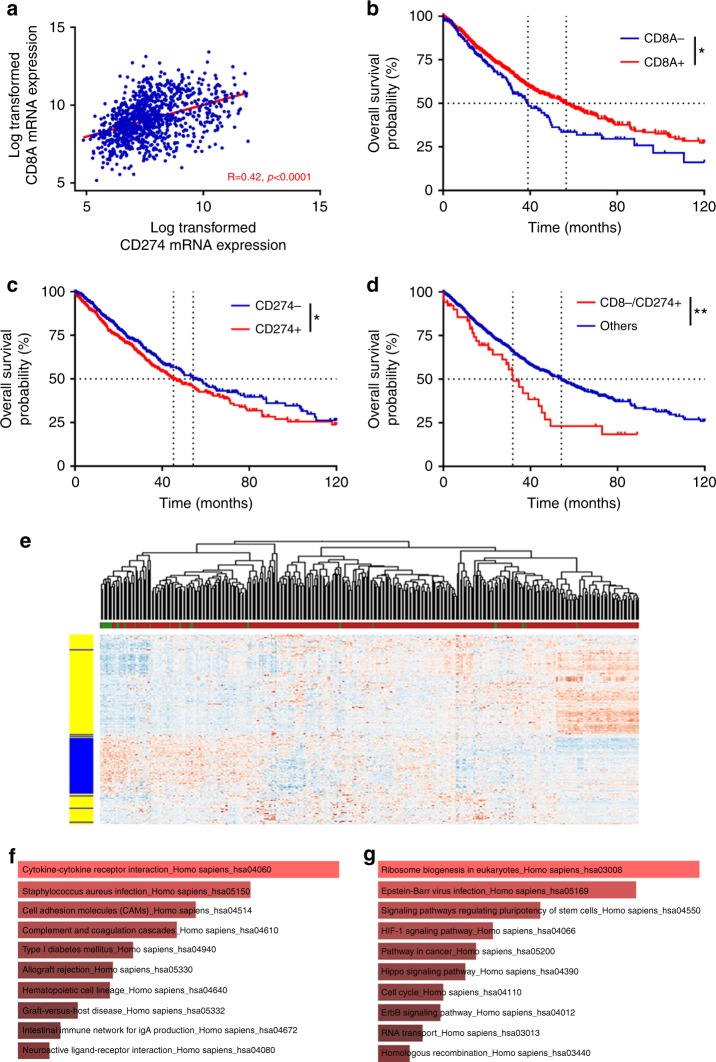
Prognostic role of CD8 and PD-L1 expressions in TCGA cohort. **a** Scatter plot showing the correlation between CD8 mRNA expression and CD274 mRNA expression. **b** Kaplan Meier estimates for overall survival; patients were stratified according to CD8 (**b**) and CD274 (**c**) mRNA expression: high expression (CD8+/CD274+; in red) and low expression (CD8-/CD274−; in blue). **d** Kaplan–Meier estimates for overall survival; patients were stratified in two groups: a group with low expression of CD8A and high expression of CD274 mRNA (CD8A−/CD274+) and a group with the other patients. **e** Heat map of genes significantly differentially expressed between patients with low-mRNA CD8 and high-PD-L1 expression, and the other patients. The row side bar represents genes upregulated in the group of interest in blue and genes downregulated in yellow. **f** Pathway selected through an enrichment analysis performed on the downregulated genes using Enrichr with the KEGG 2016 database. **g** Pathway classification of the upregulated genes using Enrichr with the KEGG 2016 database. Cutoffs for low and high expressions were defined with the Cutoff Finder method. ^*^p < 0.05; ^**^p < 0.01; ^***^p < 0.001; ^****^p < 0.0001; ns not significant

We used RNA sequencing data from 501 squamous-cell lung cancer samples and 515 adenocarcinoma NSCLC samples in TCGA. We analyzed *CD8A* (for CD8 expression) and *CD274* (for PD-L1 expression). *CD8A* and *CD274* expression were poorly correlated (Pearson’s *r* = 0.42, *p* < 0.0001) (Fig. 2a), and we observed that *CD8A* and *CD274* expression did not differ according to histological type (squamous-cell versus nonsquamous cell), tumor stage, age, or sex (Supplementary Figures 3A–D). Moreover, late stage tumors and advanced age were associated with poor outcomes. We evaluated the prognostic role of *CD8A* and *CD274* expression on OS. We observed that high *CD8A* expression was associated with better OS (56.56 months versus 39.02 months; log-rank test *p* = 0.02) (Fig. 2b), while high *CD274* was significantly associated with poor OS (45.21 months versus 54.30 months; log-rank test *p* = 0.05) (Fig. 2c).

We observed that patients with low*-CD8A* expression and high expression of *CD274* had very poor prognostic outcomes with a median OS time of 31.9 versus 54.20 months for other patients (log-rank test *p* = 0.009) (Fig. 2d). Differential gene expression analysis was performed to select genes highly or poorly expressed in this category of patients (low CD8A/high CD274). We observed that 6091 genes were downregulated in the group of patients classified as low CD8A and high CD274 compared to the whole group. A pathway analysis using KEGG showed enrichment in activated immune response, suggesting that this type of tumor is characterized by poor immune activation. In the same group of patients, we observed 2999 highly expressed genes. The analysis also showed enrichment of stemness pathways, suggesting aggressiveness and epithelial–mesenchymal differentiation of the tumor contingent (Fig. 2e–g).

The multivariate analysis showed that patients with stage II (HR = 1.53), stage III (HR = 2.11) and IV (HR = 3.09), advanced age (HR = 1.015), and high CD274 expression had a poorer prognosis, whereas high CD8A expression was associated with a better prognosis (Supplementary Table 6). The Harrell’s C statistics of this model (0.622) indicates a strong potential to discriminate patients with poor and good OS. When bootstrapping was performed to check the validity of the multivariate Cox model, CD8A and CD274 expression remained significant (CD8A *p* = 0.003 and CD274 *p* = 0.02).

In the subgroup analysis of stage III–IV patients, high CD8A expression remained associated with better outcomes while CD274 was not associated with prognosis (Supplementary Figures 3E, F). Similar to the whole population with all stages of cancer, patients with low-CD8A expression and high-CD274 expression had a poorer prognosis (Supplementary Figure 3G).

### Predictive role of CD8 TILs and PD-L1 expression in a cohort of patients treated with nivolumab

**Fig. 3:**
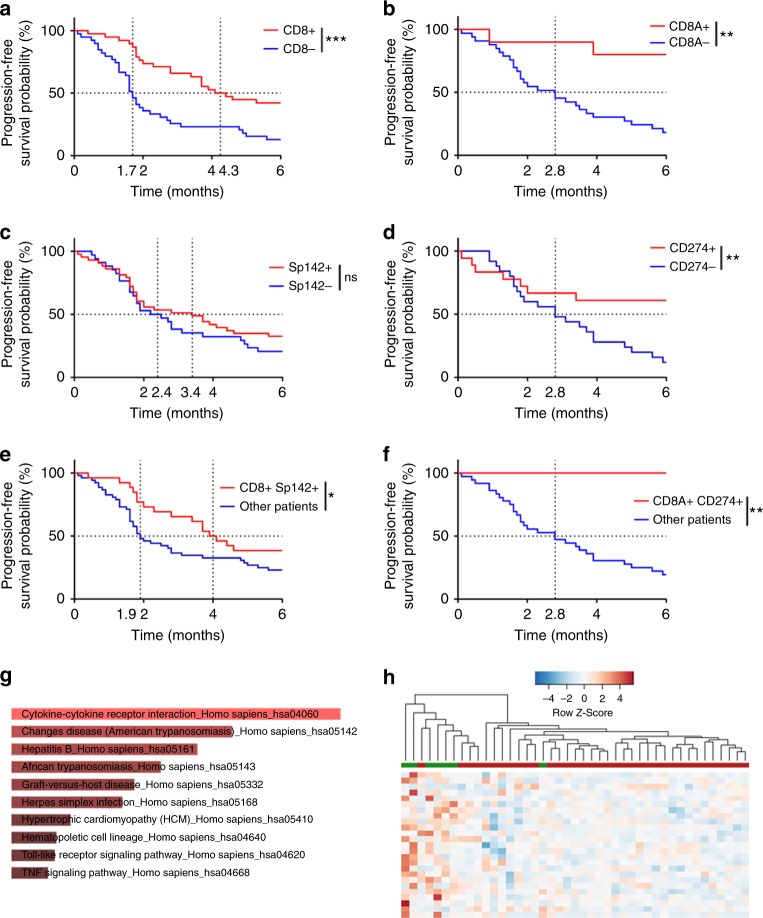
Predictive role of CD8 and PD-L1 expression in a cohort of patients treated with nivolumab. **a**, **b** Kaplan–Meier estimates for progression-free survival; patients were stratified according to CD8 IHC (A) and mRNA (**b**) expression: high expression (CD8+/CD8A+; in red) and low expression (CD8-/CD8A−; in blue). **c**, **d** Kaplan–Meier estimates for progression-free survival; patients were stratified according to PD-L1 IHC (**c**) and mRNA (**d**) expression: high expression (Sp142+/CD274+; in red) and low expression (Sp142−/CD274−; in blue). **e**, **f** Kaplan–Meier estimates for progression-free survival; patients were stratified in two groups: a group with high CD8 and PD-L1 IHC (**e**) and mRNA (**f**) expression (IHC: CD8+ Sp142+; mRNA: CD8A+ CD274+), and a group with the other patients. **g** Pathway selected through an enrichment analysis performed on the upregulated genes using Enrichr with the KEGG 2016 database. Heat map of genes significantly differentially expressed between patients with high mRNA CD8 and PD-L1 expressions and the other patients. ^*^p < 0.05; ^**^p < 0.01; ^***^p < 0.001; ^****^p < 0.0001; ns not significant

We then addressed the role of CD8^+^ TILs and PD-L1 expression to predict response to nivolumab in a cohort of 85 patients treated with nivolumab in second line or beyond. We observed that a high expression of CD8^+^ TILs measured with IHC and mRNA was significantly associated with RR and PFS (IHC: 4.3 versus 1.7 months; log-rank test *p* = 0.0002; mRNA: NR versus 2.8 months; log-rank test *p* = 0.002; Fig. 3a, b and S4A, B). For PD-L1, neither PD-L1 IHC nor mRNA expression were associated with RR (Supplementary Figures 4C, D). There was no significant association between PD-L1 expression measured by IHC on PFS (3.4 versus 2.4 months; log-rank test *p* = 0.31) (Fig. 3c). In contrast, high *CD274* expression was associated with better PFS (NR versus 2.8 months, log-rank test *p* = 0.009) (Fig. 3d). When combining these dichotomous markers, we observed that patients with high CD8^+^ TILs and PD-L1 by IHC or high *CD8A* and *CD274* coexpression had longer PFS (IHC: 4 versus 1.9 months, log-rank test *p* value = 0.04; mRNA: NR versus 2.8 months log-rank test *p* = 0.001) (Fig. 3e, f).

Using multivariate Cox proportional hazards models including age, sex, WHO performance status, and histology, we showed that CD8^+^ TILs expression measured through IHC or mRNA and PD-L1 in mRNA (but not PD-L1 determined upon IHC) remained independent predictive factors of PFS (Table 1). Harrell’s *C*-statistics indicated better discrimination for each multivariate model that included PD-L1 or CD8^+^ TILs variables for predicting PFS than a simple clinical model including sex, age, WHO performance status, and histology. Moreover, multivariate models including both CD8 and PD-L1 expression (for IHC or mRNA) were more discriminant, with an increase in Harrell’s *C*-statistic and a decrease in AIC. We noticed that for IHC variables, PD-L1 expression did not really improve the ability of CD8^+^ TILs expression to discriminate good and poor responders patients (Harrell’s *C*-statistic = 0.76 for the model with clinical variables plus CD8 IHC variable versus Harrell’s *C*-statistic = 0.78 for the model with clinical variables plus CD8 and PD-L1 IHC variables). More importantly, the mRNA model outclassed the IHC model (Harrell’s *C*-statistic: 0.78 versus 0.90) although there were fewer patients. When bootstrapping was performed to check the stability of the multivariate Cox model, the association of *CD8A* and *CD274* expression values remained significantly related to PFS (CD8A *p* = 0.048 and CD274 *p* = 0.07).

**Table 1:** Summary of univariate and multivariate Cox models built on the predictive cohort data

Multivariate Cox models																														
Variable		Univariate Cox models				Clinical model			Immunohistochemistry models						RNA sequencing models						Combined CD8/PD-L1 models									
									CD8			Sp142			CD8A			CD274			IHC			*Bootstrapping*		RNA-Seq			*Bootstrapping*	
		Hratio	95% IC	*p*	Adjusted *p* (FDR)	Hratio	95% IC	*p*	Hratio	95% IC	*p*	Hratio	95% IC	*p*	Hratio	95% IC	*p*	Hratio	95% IC	*p*	Hratio	95% IC	*p*	95%CI	*p*	Hratio	95% IC	*p*	95%CI	*p*
Sex	Female	1				1			1			1			1			1			1					1				
	Male	0.586	[0.331–1.04]	0.06	0.09	0.945	[0.489−1.826]	0.87	0.954	[0.486–1.874]	0.89	0.908	[0.454–1.815]	0.78	0.413	[0.152–1.124]	0.08	0.330	[0.114–0.954]	**0.04**	0.95	[0.481–1.859]	0.87	[0.413–2.602]	0.49	0.302	[0.105–0.868]	**0.03**	[0.037–1.555]	0.051
Age	Continuous	0.966	[0.941–0.992]	**0.01**	**0.02**	0.968	[0.942−0.994]	**0.02**	0.971	[0.943;0.999]	**0.046**	0.968	[0.941–0.996]	**0.02**	0.963	[0.926–1.002]	0.06	0.956	[0.916–0.998]	**0.04**	0.971	[0.943–1.000]	**0.047**	[0.929–1.005]	**0.037**	0.951	[0.910–0.994]	**0.02**	[0.838–1.004]	**0.034**
WHO performance status	0	1				1			1			1			1			1			1					1				
	1	0.986	[0.576–1.688]	0.96	0.96	0.879	[0.497−1.554]	0.66	0.830	[0.459–1.499]	0.536	0.875	[0.479–1.596]	0.66	0.705	[0.302–1.643]	0.42	0.850	[0.375–1.924]	0.70	0.837	[0.462–1.514]	0.56	[0.486–1.764]	0.44	0.665	[0.291–1.524]	0.34	[0.204–2.715]	0.40
	2	9.045	[3.373–24.25]	**1.2E−5**	**1.1E−4**	7.289	[2.614−20.323]	**0.0001**	5.064	[1.667–15.382]	**0.004**	6.980	[2.287–21.301]	**0.0006**	4.588	[1.137–18.515]	**0.03**	5.130	[1.283–20.521]	**0.02**	5.071	[1.657–15.522]	**0.004**	[2.189–42.804]	**0.005**	3.985	[0.993–15.988]	0.051	–	**–**
Histology	Nonsquamous cell	1				1			1			1			1			1			1					1				
	Squamous cell	0.676	[0.405–1.127]	0.13	0.18	0.812	[0.455−1.449]	0.48	0.913	[0.507–1.644]	0.76	0.823	[0.449–1.507]	0.53	1.050	[0.454–2.429]	0.91	1.256	[0.503–3.138]	0.63	0.885	[0.487–1.606]	0.69	[0.365–1.934]	0.36	1.301	[0.536–3.156]	0.56	[0.120–5.294]	0.34
CD8 expression	Low	1				-			1			-			-			-			1					-				
	High	0.375	[0.218−0.646]	0.0002	***0.0009***				0.413	[0.234−0.732]	***0.002***										0.437	[0.243−0.785]	**0.006**	[0.176−0.840]	**0.008**					
**PD-L1 expression (Sp142)**	Low	1				-			-			1			-			-			1					-				
	High	0.764	[0.452−1.292]	*0.31*	*0.35*							0.642	[0.372−1.109]	*0.11*							0.759	[0.436−1.320]	*0.33*	[0.366−2.341]	*0.26*					
**CD8 expression (CD8A)**	Low	1				78			-			-			1			-			-					1				
	High	0.14	[0.033−0.594]	**0.002**	**0.006**										0.115	[0.025−0.532]	**0.006**									0.163	[0.033−0.798]	**0.03**	[0.001−0.934]	**0.048**
**PD-L1 expression (CD274)**	Low	1				-			-			-			-			1			-					1				
	High	0.332	[0.140−0.788]	**0.009**	**0.02**													0.225	[0.079−0.638]	**0.005**						0.366	[0.127−1.051]	0.06	[0.069−1.134]	0.07
**Statistics**	AIC	-				**460**			**401**			**408**			**176**			**178**			**402**					**173**				
	Harrell’s C-statistic					**0.66**			**0.76**			**0.70**			**0.84**			**0.86**			**0.78**					**0.90**				

Differential gene expression analysis was performed to characterize highly expressed and poorly expressed genes in the category of patients with high *CD8A* and *CD274* expression. Only 26 highly expressed genes were retained. Pathway analysis using Enrichr and the 2016 KEGG database showed enrichment in activated immune response (Fig. 3g, h).

**Fig. 4:**
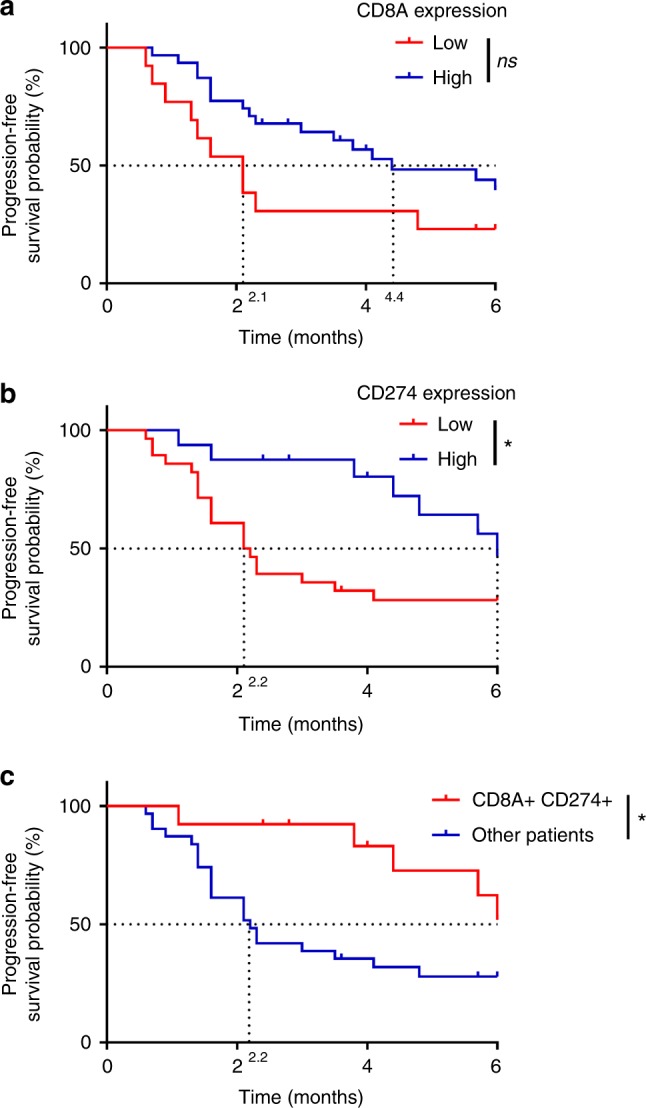
Validation of predictive role of CD8 and PD-L1 expression in a cohort of patients treated with nivolumab or pembrolizumab. **a**, **b** Kaplan–Meier estimates for progression-free survival; patients were stratified according to CD8A (**a**) and CD274 (**b**) mRNA expression: low expression (in red) and high expression (in blue). **c** Kaplan–Meier estimates for progression-free survival; patients were stratified in two groups: a group with high CD8A and CD274 mRNA expression (CD8A+ CD274+) and a group with the other patients. Cutoffs for low and high expressions were defined with the Cutoff Finder method. ^*^p < 0.05; ^**^p < 0.01; ^***^p < 0.001; ^****^p < 0.0001; ns not significant

Then, a validation of these mRNA’s results was performed from a second independent cohort of 44 patients (validation predictive cohort from Québec city, Canada). We observed that high CD8A expression was associated with a longer PFS, with a trend toward significance (median PFS 2.1 months versus 4.4 months; log-rank *p* = 0.07) (Fig. 4a). High-CD274 expression was significantly associated with a longer PFS (median PFS 2.2 versus 6 months; log-rank *p* = 0.03) (Fig. 4b). When using a combination of these dichotomous markers, we observed that patients with high *CD8A* and *CD274* expression had higher PFS (median PFS 2.2 months versus NR; log-rank *p* = 0.02) (Fig. 4c).

Our data thus underline that the coexpression CD8 and PD-L1 mRNA outperformed PD-L1 or CD8 mRNA when analyzed separately, as well as IHC variables.

### Benchmarking of CD8/PD-L1 mRNA variables in comparison with other immune signatures

**Fig. 5:**
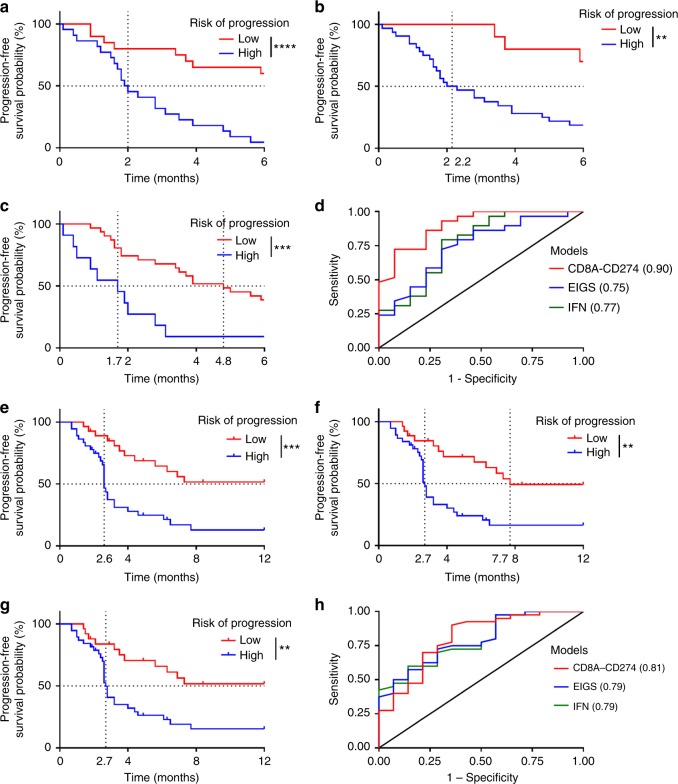
Benchmarking of CD8/PD-L1 mRNA variables in comparison with other immune signatures. **a**–**c** Kaplan–Meier estimates for progression-free survival; patients were stratified according to the value of the linear predictor estimated from the clinical Cox model plus CD8/PD-L1 mRNA (**a**), IFN signature (**b**), or EIG signature (**c**) for predictive cohort: high (blue) or low (red) risk. **d** ROC curves estimated using the linear predictor of the clinical Cox model plus CD8/PD-L1 mRNA (red), IFN signature (green), or EIG signature (blue) for predictive cohort. For each model, the area under the curve is given in brackets. **e**–**g** Kaplan–Meier estimates for progression-free survival; patients were stratified according to the value of the linear predictor estimated from the clinical Cox model plus CD8/PD-L1 mRNA (**e**), IFN signature (**f**), or EIG signature (**g**) for the extern validation cohort: high (blue) or low (red) risk. **h** ROC curves estimated using the linear predictor of the clinical Cox model plus CD8/PD-L1 mRNA (red), IFN signature (green) or EIG signature (blue) for the extern validation cohort. For each model, the area under the curve is given in brackets. Cutoffs for low and high expressions were defined with the Cutoff Finder method. ^*^p < 0.05; ^**^p < 0.01; ^***^p < 0.001; ^****^p < 0.0001; ns not significant

Using the linear predictor of the *CD8A-CD274* mRNA multivariate model as a new composite variable, the corresponding model discriminates between patients with good and poor PFS (median NR versus 2 log-rank test *p* < 0.0001) (Fig. 5a). Previous reports have established that common sets of IFN-γ– and T cell-associated inflammatory genes could predict responsiveness to PD-1 blockade across different tumor types.^16^ These signatures, made up of 6 and 18 genes, respectively, were further called IFN signature and expanded immune gene (EIG) signature. To benchmark our model, including clinical variables and *CD8A-CD274* signature, we adjusted multivariate Cox proportional hazard models including clinical variables with either IFN signature or EIG signature (Supplementary Table 7). IFN and EIG signatures did not remain significant but close to (both *p* = 0.08). Using the linear predictor of the IFN and EIG signature multivariate model as a new composite variable, these models discriminate between patients with good and poor PFS, but with less significance than CD8A-CD274 signature (CD8A/CD274: NR versus 2.0; log-rank test *p* < 0.0001; IFN: NR versus 2.2 months, log-rank test *p* = 0.03; EIG: 1.7 versus 4.8 months, log-rank test *p* = 0.001) (Fig. 5a–c). Moreover, both IFN and EIG signature models were outperformed by CD8A/CD274 mRNA model in term of prediction (Harrell’s *C*-statistics: IFN = 0.77 versus EIG = 0.75 versus CD8A/CD274 mRNA = 0.90) (Fig. 5d).

Finally, we used a previously described cohort of 65 patients with melanoma, lung cancer, head cancer, or neck cancer, and treated with anti-PD1 therapy (nivolumab and pembrolizumab), to perform external validation of our RNA signature in different types of cancer. The samples were analyzed on the nCounter system using the PanCancer 730-Immune Panel.^30^ In this cohort, IFN, EIG, and CD8/CD274 mRNA signatures were used to predict PFS (Supplementary Table 8) (Fig. 5e–g). We also confirmed that the CD8A*/CD274* signature had similar predictive properties than IFN or EIG signatures (Harrell’s *C*-statistics: IFN = 0.79 versus EIG = 0.79 versus CD8/PD-L1 mRNA = 0.81) (Fig. 5h). Similar results were observed in the validation predictive cohort from Quebec city (Supplementary Figure S5). Altogether, the findings demonstrate the validity of the CD8A/CD274 signature to predict PFS in another external cohort of patients treated with anti-PD-1 antibodies, thus generalizing our observation.

## Discussion

Immune checkpoint inhibition has changed anticancer strategy in solid tumors. Efforts are currently being made worldwide to get a better understanding of this immune axis and its crosstalk with the tumor microenvironment, notably with TILs. Despite an increasing number of published articles in this field, there is no clear consensus for two essential concerns. The first major concern is the effect of PD-L1 expression on NSCLC survival. The second and perhaps most important concern is to identify the biomarkers that could efficiently select patients who would benefit the most from immune intervention targeting the PD-L1/PD-1 axis.

This study underlines the predictive and prognostic role of CD8^+^ TILs and PD-L1 expression in lung cancer. The prognostic value of PD-L1 expression in solid tumors is still unclear, and past studies have yielded controversial results. In NSCLC, a high level of PD-L1 expression has been associated with poor clinical outcomes,^31–34^ although these findings remain controversial.^35,36^ A recent meta-analysis suggested that PD-L1 expression was associated with a poor prognosis in Asian population.^37^ On the contrary, it is well documented that CD8^+^ TILs are positively correlated with a better prognosis in patients suffering from NSCLC.^2,31,38^ No study analyzed both CD8^+^ TILs and PD-L1 as prognostic factor.

The first part of our study was dedicated to the evaluation of PD-L1 and CD8^+^ T cells as prognostic markers in NSCLC. With two cohorts and two different methods (i.e., RNA sequencing and IHC procedures), we confirmed that CD8 expression was associated with a good prognosis, whereas PD-L1 did not prove to be a valuable prognostic biomarker even if a trend was observed. Subsequently, we tested the prognostic role of the four Teng’s immunological groups and observed that intrinsic induction group (PD-L1^high^/CD8^+^ TILs^low^ group) determined upon IHC and mRNA is associated with a poorer prognosis than the three other groups. This is observed in both early and late stage NSCLC. In a molecular point of view this intrinsic induction group was characterized by a loss of immune genes and an increase in stemness and genes that increase tumor aggressiveness. Our results clearly underscored the advantage of combining these two variables to classify patients according to their prognosis.

Even though immune checkpoint inhibitors (ICI) play a major role in the management of NSCLC, the absence of biomarkers remains a major issue, and only 20–30% of patients treated with ICI achieved clinical benefit. We focused on this aspect in the second part of our study.

At present, the evaluation of PD-L1 expression upon IHC in NSCLC is the only available biomarker in clinics, but there are several limitations. It is now well accepted that PD-L1 expression is heterogeneous in the tumors of most types of cancer. A recent study showed a discordance in PD-L1 assessment between biopsy and surgically resected specimens.^39,40^ In NSCLC, the analysis is generally performed on a biopsy, which does not reflect the expression of PD-L1 in the whole tumor. Furthermore, PD-L1 assessment with IHC procedures is done using different anti-PD-L1 antibodies and cut-off criteria. In different published studies, the mAbs targeting PD-L1, the methods of analysis and the threshold for PD-L1 positive expression with IHC procedures were different. In an effort to harmonize PD-L1 IHC assessment, a blueprint project assessed interobserver concordance^41^ and demonstrated that the result of PD-L1-stained tumor cells was comparable using 22C3, 28-8, and SP263 clones, whereas the SP142 assay exhibited fewer stained tumor cells. However, in the context of NSCLC 22C3 and SP142 share similar power to predict response to checkpoint inhibitors.^42^ However, PD-L1 positivity alone cannot be considered as an optimal factor for predicting response to PD-1/PD-L1 blockade.

Some studies have reported an association between PD-L1 expression and CD8^+^ TILs density in lung cancer.^34,43^ Few studies have examined CD8 expression as a predictive biomarker.^44^ A recent study published in Nature described CD8 profiling in the peripheral blood of patients with melanoma treated with ICI. The findings demonstrated an association between CD8 activation and ICI response.^45^ Tumeh et al.^19^ and Le et al.^46^ demonstrated an association between CD8 expression in tumors upon IHC and response to ICI in melanoma and colorectal cancer, respectively. Based on these results, our intention was to evaluate CD8 expression as a predictive biomarker of response to nivolumab in NSCLC.

Firstly, using the same methodology as our prognostic cohorts, we demonstrated that IHC evaluation of CD8^+^ TILs alone was a powerful biomarker that discriminated patients receiving immune checkpoint inhibitors for PFS. The study of PD-L1 expression with IHC procedures did not yield additional information. Even if CD8^+^ TILs evaluation with IHC procedures is typically more robust than PD-L1 evaluation, we chose to use a semiautomated method to accurately count CD8^+^ T cells. The automatization of this type of quantitative analysis using open-source software is less time consuming and more informative than standard evaluation by a pathologist. Automated analysis should be more widely used to homogenize published results.

Secondly, CD8A and CD274 mRNA quantification using RNAseq was predictive to ICI response. Interestingly, the combination of the two factors outperformed the discriminatory properties of CD8A or CD274 variables alone, as well as CD8^+^ TILs and PD-L1 IHC variables. Moreover, we observed that the CD8A/CD274 two-genes signature was superior to two previously published gold-standard signatures.^16^ The CD8A/CD274 signature was also validated in a public data set which involves NSCLC, head and neck, melanoma treated with anti PD1 or PD-L1 mAb. In addition, we also validated this biomarker in an additional cohort of metastatic lung cancer patients from Quebec City treated with pembrolizumab in first line or nivolumab in further line. We believe that our data support the rational that CD8^+^ TILs infiltration and expression of PD-L1 are both required for the efficacy of checkpoint inhibitors targeting PD-1 or PD-L1 in lung cancer treated with immunotherapy in first or further line. This intuitive result corroborates the proposal of tumor classification according to CD8^+^ TILs and PD-L1 level by Teng’s.^20^ Furthermore, our signature outperformed other previous signatures in an external cohort with various tumor types. These data underline the stability of the model, making our observations relevant for other tumor types, other anti-PD-1 antibodies and other technologies.

In conclusion, our retrospective study showed for the first time that combining CD8^+^ TILs and PD-L1 assessment seems to outperform CD8^+^ TILs or PD-L1 alone as a prognostic marker in NSCLC and as predictive signature of ICI response. Transcriptomic data enabled us to pull out a CD8A/CD274 two-genes signature that identified good responders. In exploratory analysis, this signature outperformed recently published extended immune and interferon signatures and yields better results than IHC data. Limitations of our study include the retrospective design of the study and the low number of patients, so our findings need to be validated by prospective studies and clinical trials. If the CD8A/CD274 signature is validated by further study, this novel biomarker might be adapted to routine analysis and could be used by clinicians to address prognostic of patients and select patients eligible for ICI administration.

### Data availability

The data sets generated and/or analyzed during the current study, as well as the computer code used to perform statistical analysis, are available from https://github.com/CRichard21/CD8PDL1_Nivolumab

## Electronic supplementary material

Figure Legends

Figure S1

Figure S2

Figure S3

Figure S4

Figure S5

Supplementary Table 1

Supplementary Table 2

Supplementary Table 3

Supplementary Table 4

Supplementary Table 5

Supplementary Table 6

Supplementary Table 7

Supplementary Table 8

## REMARK Guidelines

This study conforms to REMARK guidelines.
