# Correction: Prognostic and predictive role of CD8 and PD-L1 determination in lung tumor tissue of patients under anti-PD-1 therapy

**DOI:** 10.1038/s41416-019-0512-8

**Published:** 2019-06-24

**Authors:** Jean-David Fumet, Corentin Richard, Fanny Ledys, Quentin Klopfenstein, Philippe Joubert, Bertrand Routy, Caroline Truntzer, Andréanne Gagné, Marc-André Hamel, Camila Figueiredo Guimaraes, Bruno Coudert, Laurent Arnould, Laure Favier, Aurélie Lagrange, Sylvain Ladoire, Pierre Saintigny, Sandra Ortiz-Cuaran, Maurice Perol, Pascal Foucher, Paul Hofman, Marius Ilie, Sandy Chevrier, Romain Boidot, Valentin Derangere, François Ghiringhelli

**Affiliations:** 1 Department of Medical Oncology, Center GF Leclerc, Dijon, France; 2 Research Platform in Biological Oncology, Dijon, France; 3 GIMI Genetic and Immunology Medical Institute, Dijon, France; 4 University of Burgundy-Franche Comté, Dijon, France; 5 Institut Universitaire de Cardiologie et de Pneumologie de Québec, Laval University, Quebec City, QC Canada; 6 Centre de Recherche du Centre Hospitalier de l’Université de Montréal (CRCHUM), Montréal, QC Canada; 7 Hematology–Oncology Division, Department of Medicine, Centre Hospitalier de l’Université de Montréal (CHUM), Montréal, QC Canada; 8 Department of Pathology, Center GF Leclerc, Dijon, France; 9 Department of Medical Oncology, Center Leon Berard, Lyon, France; 10 Department of Thoracic Oncology, Dijon University Hospital, Dijon, France; 11 Laboratory of Clinical and Experimental Pathology, FHU OncoAge, Nice University Hospital, Université Côte d’Azur, Nice, France; 12 Hospital-Integrated Biobank (BB-0033-00025), FHU OncoAge, Nice University Hospital, Université Côte d’Azur, Nice, France; 13 INSERM UMR1231, Dijon, France

**Keywords:** Predictive markers, Tumour biomarkers

**Correction to:** *British Journal of Cancer* (2018) **119**, 950–960; 10.1038/s41416-018-0220-9; published online 15 October 2018

Since the publication of this paper, the authors noticed that Corentin Richard was not credited as contributing equally to the paper. He should be considered as a joint first author with Jean-David Fumet.

