# Supplementary material for: Prognostic and predictive role of CD8 and PD-L1 determination in lung tumor tissue of patients under anti-PD-1 therapy
Source: Br J Cancer. 2018 Oct 15;119(8):950–60. doi: 10.1038/s41416-018-0220-9 (PMC6203820; doi:10.1038/s41416-018-0220-9)
Supplement: Supplementary file 1 — Figure Legends [file 41416_2018_220_MOESM1_ESM.docx]

Figure S1:

(A) (Upper panel) PD-L1 staining in percentage of positive tumor cells (TC) where 0 is lower than 1%, 1 is between 1% and 5%, 2 between 5% and 50% and 3 is higher than 50%. (Lower panel) PD-L1 staining in percentage of positive immune cells (IC) where 0 is lower than 1%, 1 is between 1% and 5%, 2 is between 5% and 10% and 3 is higher than 10% (lower panel). Yellow bar scale at the bottom indicates 50µm. (B) Semi-automated evaluation of CD8^+^ cells using QuPath software. Cells were counted in areas within red boundaries. CD8^+^ positive cells are displayed with red contours while negative cells are displayed with blue contours. (Upper panel) Representative snapshots of CD8^+^ low patient. (Lower panel) Representative snapshots of CD8^+^ high patient. Yellow scale bar is 500µm for low magnification and 100µm for high magnification.

Figure S2:

(A) Representative snapshots of typical low (upper panel) and high CD8 staining (lower panel) at low and high magnification. Scale bar is respectively 500µm and 100µm. (B) Representative snapshots of the same sample stained with Sp142 and 22C3 anti-PD-L1 clones. Scale bar is 250µm. (C) Cumulative bar plots representing the proportion of patients with high Sp142 labeling expression (Sp142+; light grey) or low Sp142 labeling expression (Sp142-; black), respectively for patients with a 22C3 labeling expression < 1%, between 1 and 49% and ≥ 50%.

Figure S3:

(A-D) Box plots showing the mRNA expression of CD8A and CD274 genes comparing distribution between histological classifications (A), tumor stage (B), age (C) and sex (D). (E-F) Kaplan-Meier estimates for overall survival; patients with stage III or IV were stratified according to CD8 (E) and CD274 (F) mRNA expression: high expression (CD8+/CD274+; in red) and low expression (CD8-/CD274-; in blue). (G) Kaplan-Meier estimates for overall survival; patients with stage III or IV were stratified in two groups: a group with low CD8 and high CD274 mRNA expression (CD8-/CD274+) and a group with the other patients. Cutoffs for low and high expressions were defined with the Cutoff Finder method. *: *p < 0.05*; **: *p < 0.01*; ***: *p < 0.001*; ****: *p < 0.0001*; *ns*: not significant.

Figure S4:

(A-B) Cumulative bar plots showing the proportion of patients with high CD8 expression (CD8+/CD8A+; light grey) or low CD8 expression (CD8-/CD8A-; black) for IHC (A) and mRNA (B) analyses, respectively for patients with progression-disease RECIST response (PD) and pooled stable-disease and partial response (SD + PR). (C-D) Cumulative bar plots showing the proportion of patients with high PD-L1 expression (Sp142+/CD274+; light grey) or low PD-L1 expression (Sp142-/CD274-; black) for IHC (A) and mRNA (B) analyses, respectively for patients with progression-disease RECIST response (PD) and pooled stable-disease and partial response (SD + PR). Cutoffs for low and high expressions were defined with the Cutoff Finder method. *: *p < 0.05*; **: *p < 0.01*; ***: *p < 0.001*; ****: *p < 0.0001*; *ns*: not significant.
