# Supplementary figures and images for: Prognostic and predictive role of CD8 and PD-L1 determination in lung tumor tissue of patients under anti-PD-1 therapy
Source: Br J Cancer. 2018 Oct 15;119(8):950–60. doi: 10.1038/s41416-018-0220-9 (PMC6203820; doi:10.1038/s41416-018-0220-9)

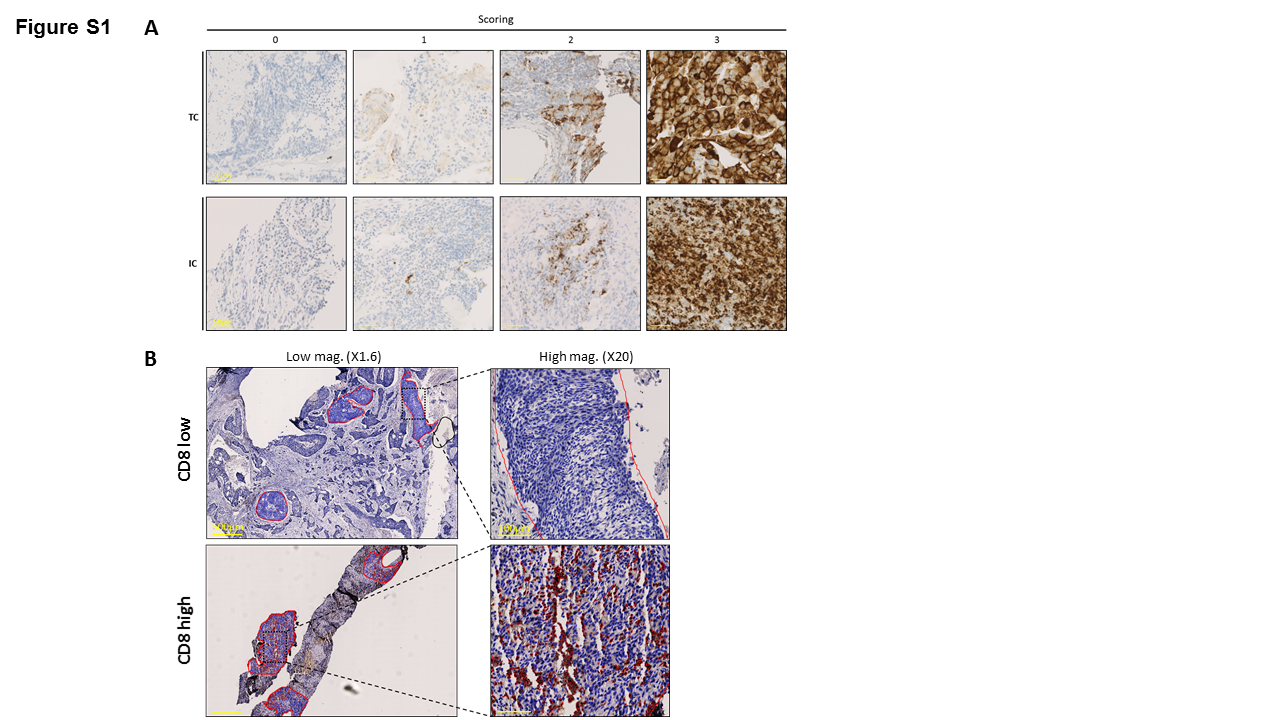

Supplement: Supplementary file 2 — Figure S1 [file 41416_2018_220_MOESM2_ESM.tif]

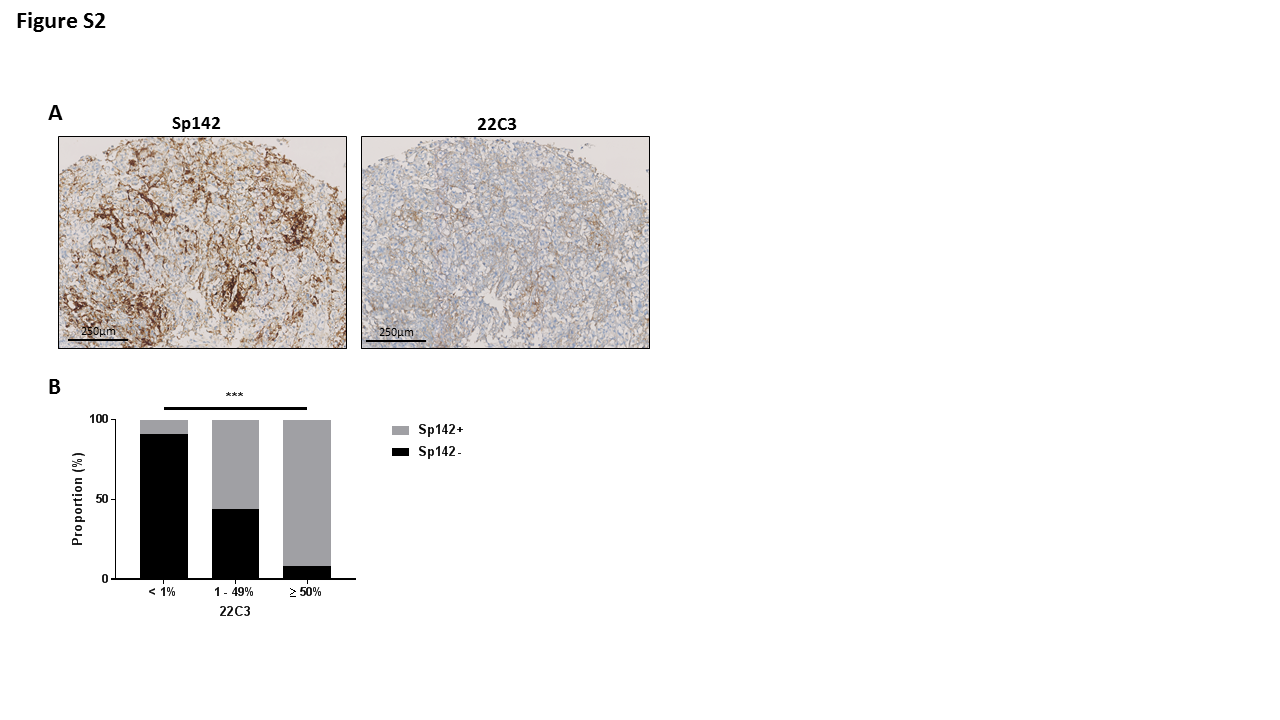

Supplement: Supplementary file 3 — Figure S2 [file 41416_2018_220_MOESM3_ESM.tif]

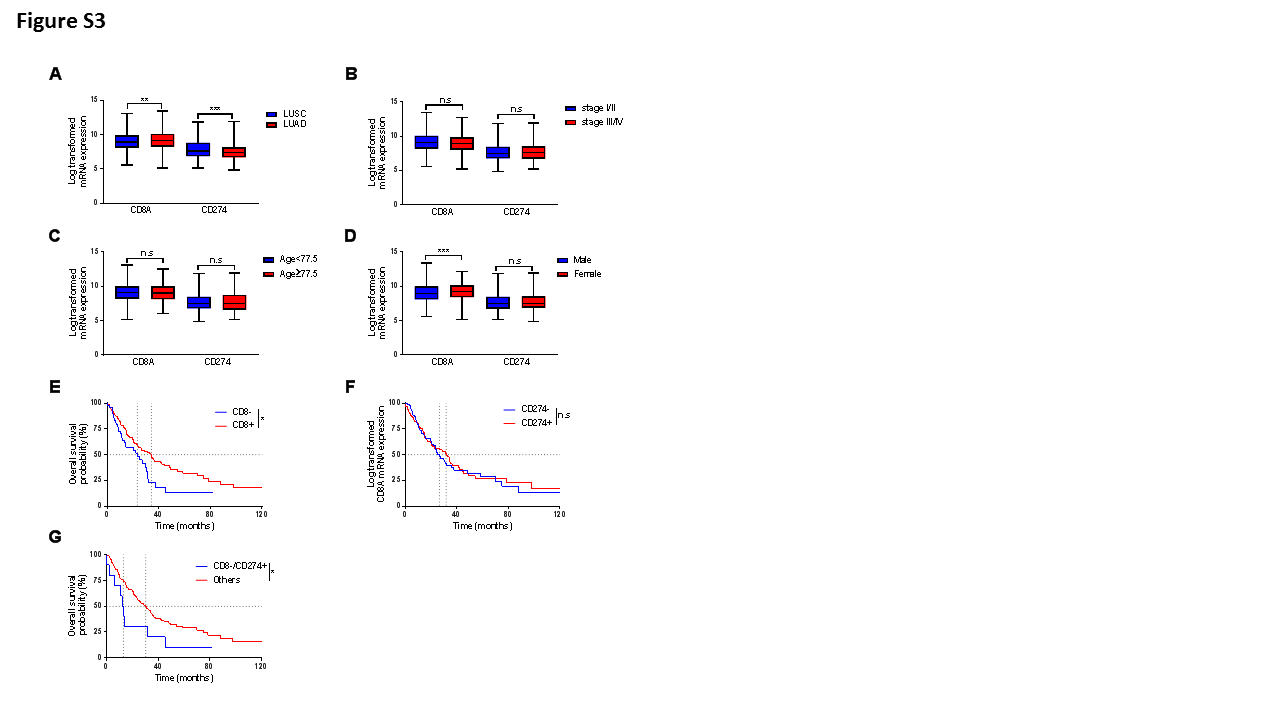

Supplement: Supplementary file 4 — Figure S3 [file 41416_2018_220_MOESM4_ESM.tif]

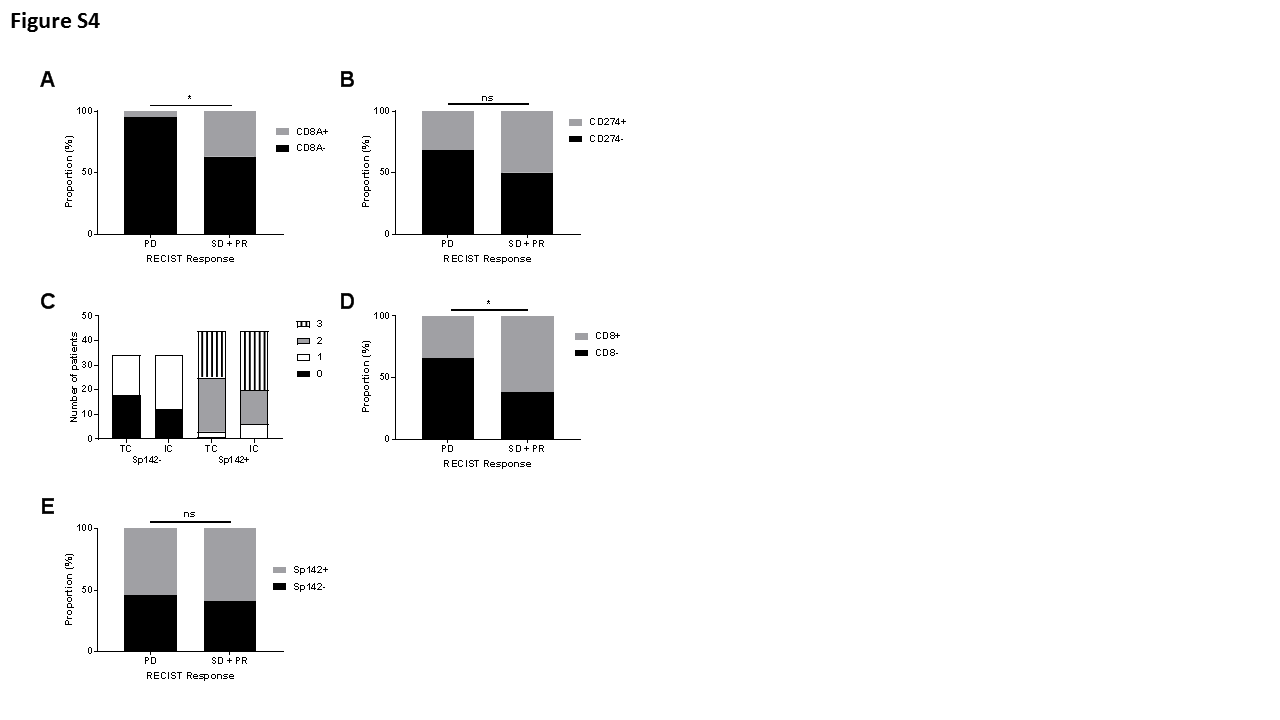

Supplement: Supplementary file 5 — Figure S4 [file 41416_2018_220_MOESM5_ESM.tif]

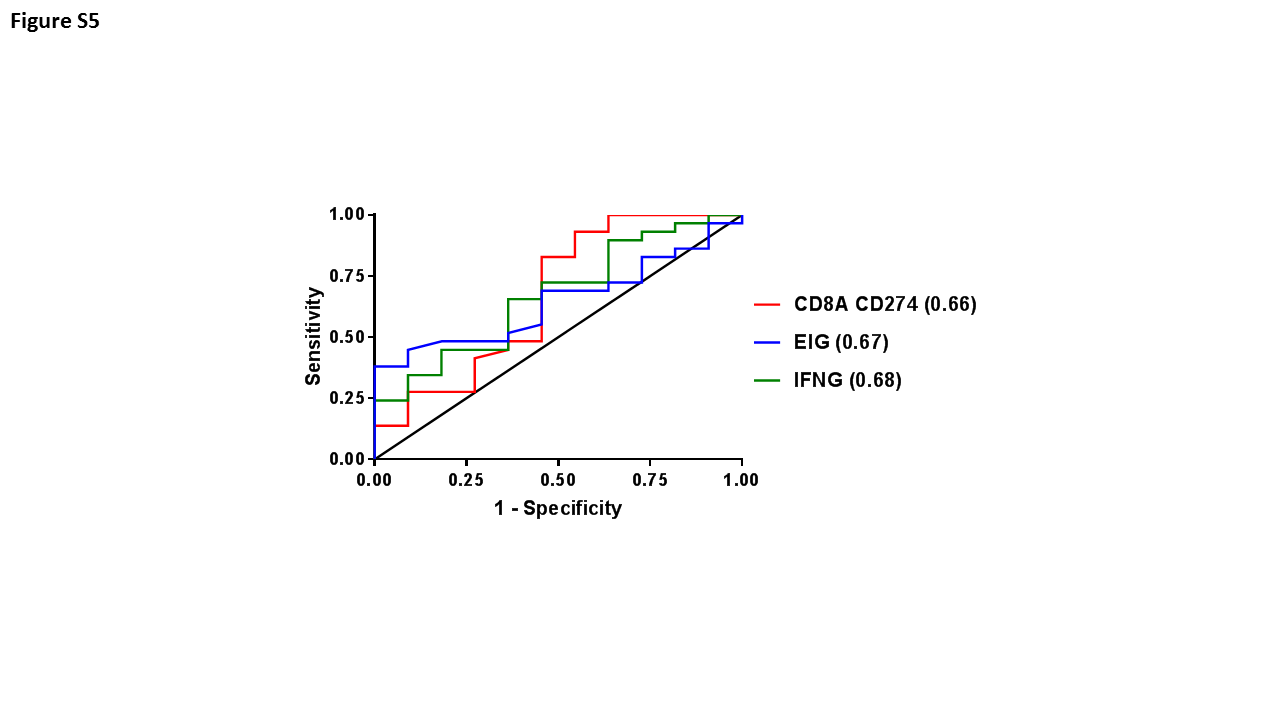

Supplement: Supplementary file 6 — Figure S5 [file 41416_2018_220_MOESM6_ESM.tif]
